# NUT Carcinoma: Clinicopathologic Features, Molecular Genetics and Epigenetics

**DOI:** 10.3389/fonc.2022.860830

**Published:** 2022-03-16

**Authors:** Vanessa Moreno, Karan Saluja, Sergio Pina-Oviedo

**Affiliations:** ^1^ Department of Pathology and Laboratory Medicine, McGovern Medical School, The University of Texas Health Science Center at Houston, Houston, TX, United States; ^2^ Department of Pathology, Duke University Medical Center, Durham, NC, United States

**Keywords:** NUT carcinoma, NUT midline carcinoma, BRD-NUTM1, NSD3, zinc finger proteins, BET inhibitors, HDAC inhibitors

## Abstract

Nuclear protein in testis (NUT) carcinoma is a rare, highly aggressive, poorly differentiated carcinoma occurring mostly in adolescents and young adults. This tumor usually arises from the midline structures of the thorax, head, and neck, and exhibits variable degrees of squamous differentiation. NUT carcinoma is defined by the presence of a *NUTM1* (15q14) rearrangement with multiple other genes. In about 70-80% of the cases, *NUTM1* is involved in a balanced translocation with the *BRD4* gene (19p13.12), leading to a *BRD4-NUTM1* fusion oncogene. Other variant rearrangements include *BRD3-NUTM1* fusion (~15-20%) and *NSD3-NUTM1* fusion (~6%), among others. The diagnosis of NUT carcinoma requires the detection of nuclear expression of the NUT protein by immunohistochemistry. Additional methods for diagnosis include the detection of a *NUTM1* rearrangement by fluorescence *in situ* hybridization or by reverse transcriptase PCR. NUT carcinoma is usually underrecognized due to its rarity and lack of characteristic histological features. Therefore, the goal of this review is to provide relevant recent information regarding the clinicopathologic features of NUT carcinoma, the role of the multiple *NUTM1* gene rearrangements in carcinogenesis, and the impact of understanding these underlying molecular mechanisms that may result in the development of possible novel targeted therapies.

## Introduction

Nuclear protein in testis (NUT) carcinoma (NC), is a rare carcinoma characterized by a chromosomal rearrangement involving the NUT midline carcinoma family member 1 (*NUTM1)* gene, also known as *NUT* gene, located on chromosome 15q14 (1–5). This entity was first described in 1991 in two independent case reports of mediastinal carcinomas characterized by the t(15;19) translocation (6, 7). Since then, tumors harboring *NUTM1* translocation have been increasingly recognized with numerous cases reported in the literature. Similarly, the mechanisms underlying the multiple *NUTM1* gene rearrangements have opened the door to better understand tumor pathogenesis and the role of NUT and other proteins in the epigenetics of this rare neoplasm and multiple other cancers. Here, we review the clinicopathologic features, methods of diagnosis, and the molecular genetics and epigenetic alterations known to date in NC.

## Epidemiology and Clinical Features

NC is a rare, poorly differentiated carcinoma characterized by an aggressive clinical behavior and advanced stage at diagnosis (8). Although the cell of origin is unknown, it has been speculated that NC may arise from primitive neural crest-derived cells (8). Despite lack of knowledge regarding a definitive anatomical site of origin, NC has been described to typically originate from midline structures of the thorax or from head and neck, hence the original term “NUT midline carcinoma”, and to predominantly affect young patients or adolescents. However, over the last decade, this entity has been identified in patients of all ages (0 - 81.7 years) with a median age varying from 16 to 24 years, observed in four meta-analysis studies (9–12), and affecting females and males almost equally (9, 10, 12). The most common location of primary NC has been found in the thorax (~50%) followed by the head and neck region (~40%) (12), but NC can also rarely arise outside of midline locations such as bladder (13), ocular globe (13), salivary glands (14–28), brain (29) kidney (29–31), stomach (29), adrenal gland (2, 20), pancreas (32), soft tissue (29), and bone (33).

## Histopathologic Features

**Figure 1:**
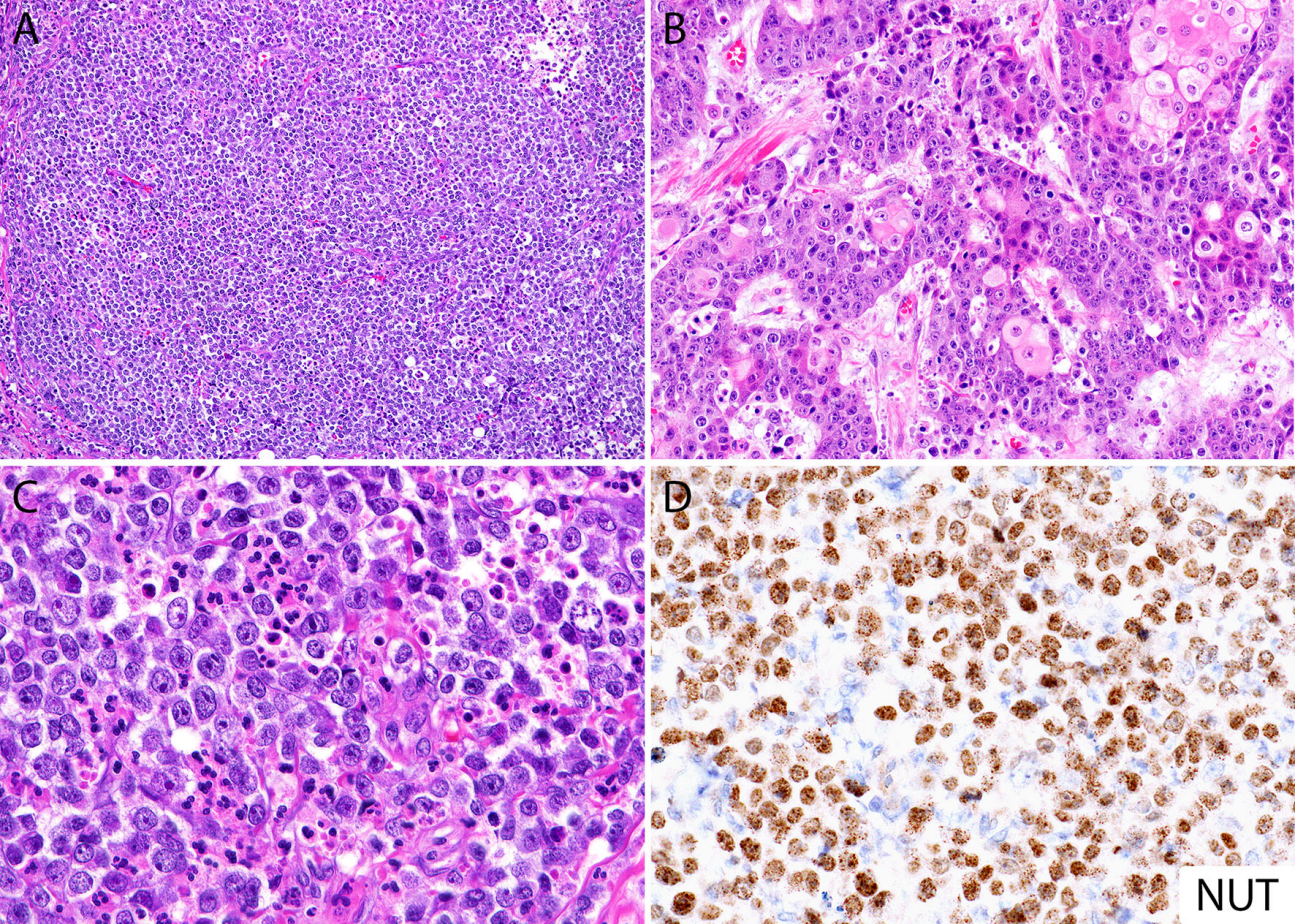
Histomorphology of NUT carcinoma and diagnosis by immunohistochemistry. **(A)** Diffuse sheets of poorly differentiated monotonous round cells with focal necrosis (top right). **(B)** Areas with abrupt keratinization in NC can be seen in up to 30% of cases. **(C)** Focal cytoplasmic clearing and intratumoral neutrophilic inflammatory infiltrate. **(D)** NUT immunohistochemical stain (monoclonal antibody, clone C52) shows diffuse nuclear labeling, often with a speckled pattern. Same case from panel **(B)**.

By histology, NC is a poorly differentiated malignant neoplasm that usually grows as nests and sheets of primitive cells without an overlying *in situ* component (34), most of them (~55%) without squamous differentiation (12), and often with areas of confluent necrosis (34–36) (**Figure 1A**). The cells may have little or moderate amount of eosinophilic or amphophilic cytoplasm with indistinct borders, imparting the appearance of a cellular syncytium (**Figures 1A–C**). Focal cytoplasmic clearing or vacuolization (**Figure 1C**) (28, 34) as well as lumen or pseudo-lumen formation have been described (34). The tumor cells can be widely infiltrative and demonstrate a high mitotic rate (34, 35). The nuclei are usually large and quite monotonous, with vesicular chromatin and distinct nucleoli, lacking the pleomorphism typically encountered in high-grade carcinomas (3, 34, 35). A peculiar feature described in the literature as a clue to the diagnosis is the presence of keratinization in the form of large cells with intercellular bridges, focal squamous “pearls” or frank abrupt keratinization (i.e., poorly differentiated cells immediately adjacent to well differentiated squamous cells) (**Figure 1B**). However, this morphologic feature is only observed in about 30% of the cases (12) and it can also be seen in HPV-associated basaloid squamous cell carcinomas (3, 35). The background stroma varies from edematous, slightly myxoid to fibrous with variable amounts of desmoplasia (14, 36). The presence of an intratumoral neutrophilic infiltrate is common and can be very prominent (**Figure 1C**), and occasionally an intraepithelial and stromal lymphocytic infiltrate may also be observed (14, 28, 37).

## Molecular Genetics and Epigenetics

The pathogenesis of NC is characterized by translocation-associated fusion oncoproteins that block cell differentiation and promote cellular growth (5). This distinct feature of single chromosomal translocation resembles those found in hematopoietic and mesenchymal malignancies and distinguishes NC from other epithelial tumors where multiple sequential mutations are required for tumorigenesis (38).

**Figure 2:**
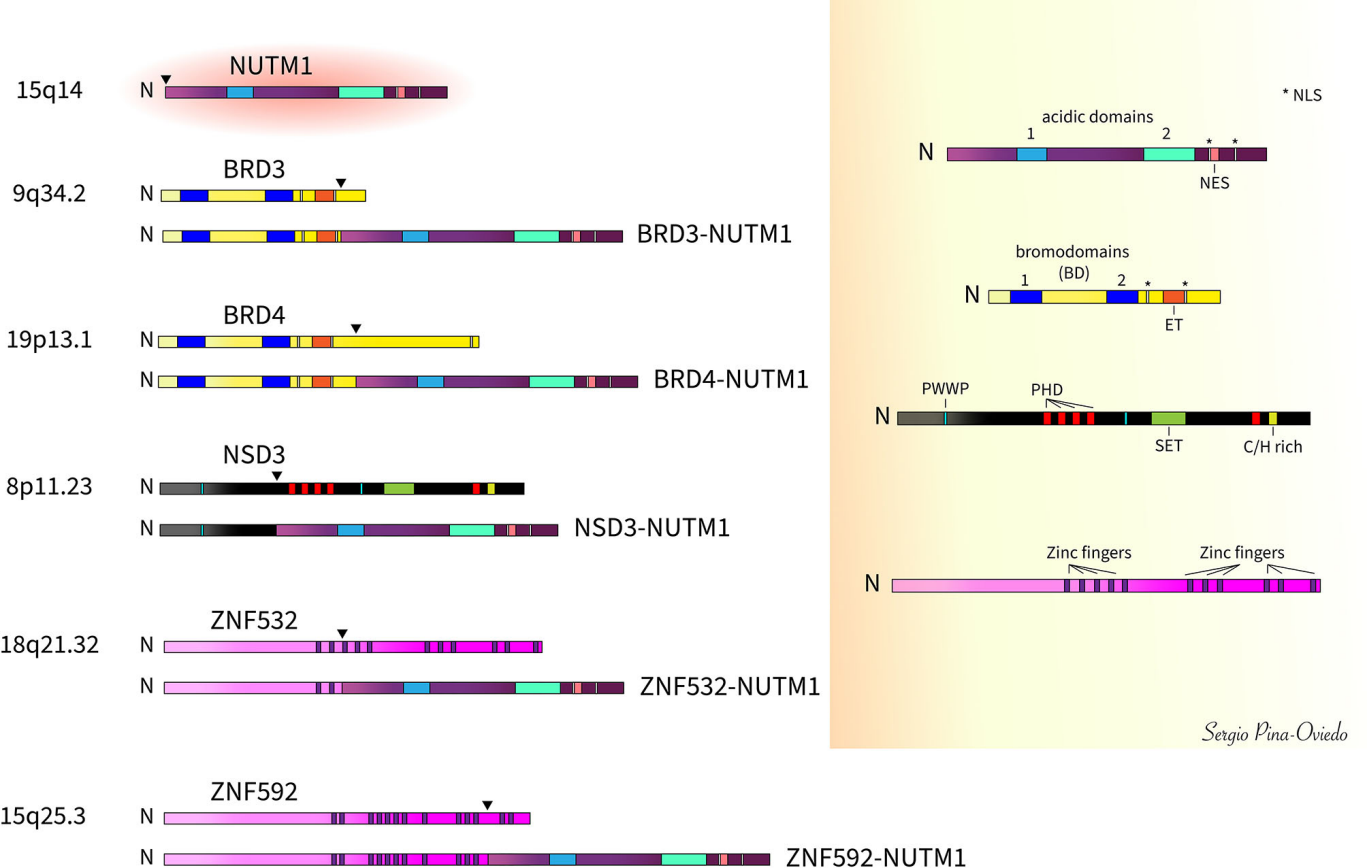
Schematic of *NUTM1* fusions with *BRD3*, *BRD4*, *NSD3*, *ZNF532*, and *ZNF592* and respective wild-type proteins (arrowheads denote fusion breakpoints). The most common of these fusions is *BRD4-NUTM1* comprising about 70-80% of all cases. [N, amino- or N-terminal; NLS, nuclear localization signal; NES, nuclear export signal; BD, bromodomains (BD1 and BD2); ET, extra-terminal domain; PWWP, Proline-Tryptophan-Tryptophan-Proline domain; PHD, plant homeo-domain-type zinc-finger motifs; SET, Su(var)3-9, Enhancer-of-zeste and Trithorax (SET) domain; C/H rich, SET-associated Cys-His-rich (SAC) domain].

NC is defined by the rearrangement of *NUTM1* gene on chromosome 15q14, which is frequently fused to the bromodomain containing protein 4 (*BRD4*) gene on chromosome 19p13.12, resulting in the most characteristic reciprocal translocation t(15;19) observed in 70-88% of the cases (9–13, 39). The predominant oncogenic variant involves the in-frame fusion of *BRD4* exon 11 to the start of *NUTM1* exon 2 (4, 6, 40, 41). However, variations of the specific exon fusions are known to occur including *BRD4* exon 11 to *NUTM1* exon 1b (41), *BRD4* exon 15 to *NUTM1* exon 2 (41), and *BRD4* exon 15 to a partially deleted *NUTM1* exon 2 (fusion at the last 124 nucleotides of *NUTM1* exon 2) (42). The *BRD4-NUTM1* fusion gene contains nearly the whole coding region for *NUTM1* (exons 1b/2 to 7) and the three well characterized domains of *BRD4* including the two bromodomains (BD 1 and BD2) and the extra-terminal (ET) domain, and a bipartite nuclear localization sequence (NLS) (4, 5). Its carboxyl-terminal domain (CTD), known to interact with the core positive transcription elongation factor b (P-TEFb), is absent (43) (**Figure 2**). The *NUTM1* gene encodes an unstructured protein with two acidic transcriptional activation domains (AD1 and AD2), a NLS, and a nuclear export signal (NES) (5) (**Figure 2**). About 15-30% of NCs have a variant translocation involving exon 9 of the bromodomain containing protein 3 (*BRD3*) gene on chromosome 9q34.2 (5, 12). *BRD3* gene encodes a protein similar to BRD4. The in-frame fusion involving *BRD3* partner gene includes almost the entire *NUTM1* structure (exons 2 to 7) along with the dual BDs, ET domain and the bipartite NLS of *BRD3* (5) (**Figure 2**).

Other rarer *NUTM1* fusion variants have been reported in about 6% of cases (12) including the nuclear receptor binding SET domain protein 3 (*NSD3*) gene (38), the zinc finger-containing protein encoding genes *ZNF532* (44) and *ZNF592* (45), and other yet unknown genes. The *NSD3* gene is located on chromosome 8p11.23 and is considered a BET-binding protein. The *NSD3* portion of the fusion (exons 1 to 7) lacks the Su(var)3-9, Enhancer-of-zeste and Trithorax (SET) domain and contains only one of the two Proline-Tryptophan-Tryptophan-Proline motif (PWWP) domains, whereas nearly all *NUTM1* (exons 2 to 7) is included in the fusion (38) (**Figure 2**). The *ZNF532-NUTM1* fusion gene encodes only the first 2 of 12 zinc finger domains from *ZNF532* and almost the entire *NUTM1* coding sequence (part of intron 1 of *NUTM1* and its remaining exons 2 to 7) (44) (**Figure 2**). The first zinc finger included in the *ZNF532-NUTM1* fusion gene encodes a putative zinc-ribbon domain that is predicted to bind nucleic acids directly (44). The ZNF592-NUTM1 resultant fusion protein contains the coding sequence of *ZNF592* up to exon 10 fused with exons 2 to 10 of *NUTM1*. The ZNF592 moiety of the fusion protein retains the first 11 of 13 zinc finger domains (45) (**Figure 2**).

In a subset of malignant solid tumors from soft tissue and other organs, of uncertain relationship to NCs, *NUTM1* has been reported to be fused with *YAP1* (46, 47), *MXD1* (29), *MXD4* (39, 48, 49), *CIC* (50, 51), *BCORL1* (29), *ATXN1* (52), and *MGA* (39, 53, 54), in which most of them have been described to occur within the context of histologically defined high-grade sarcomas likely to be associated with a distinct pathogenetic pathway.

### Bromodomain and Extra-Terminal (BET) Proteins

The BRD4 protein, encoded by the *BRD4* gene, is the most extensively studied member of the bromodomain and extra-terminal (BET) protein family (i.e., BRD2, BRD3, BRD4, and BRDT). BET proteins bind to transcriptionally active chromatin through associations of one of their bromodomains (BD1 or BD2) to acetyl-lysin residues of histones (H3 and H4) affecting cell cycle progression and cellular proliferation (55). In cancer, BET BDs promote M to G1 cell cycle progression (56). These BDs are also responsible for tethering of the BRD4-NUTM1 to chromatin (5, 57). In addition, BET proteins activate transcription of target genes through recruitment of the multiprotein Mediator complex and P-TEFb, in which the Mediator complex is a transcriptional coactivator and the P-TEFb is a cyclin dependent kinase (CDK) containing the catalytic subunit CDK9 and one of several regulatory cyclin subunits (cyclin T1, T2, and K), thereby enhancing transcriptional elongation through phosphorylation of RNA Polymerase II on serine 2 of its CTD (58, 59). Although the CTD of the BRD4 is absent in the BRD4-NUTM1 fusion protein, the BD2 plays an important role binding P-TEFb and also interacting with wild-type BRD4 and other BRDs through the N-terminal portions of BRD4 representing, most likely, the required factors for the transcriptional activating function of *BRD4-NUTM1* oncogene (43, 44). The ET domain of BET proteins is a protein-protein interaction module that binds NSD1-3, and other mediator components (43). BRD3/BRD4 also bind strongly to the regulatory regions of E2F1 transcriptional targets to enhance their activation; hence, BRD3/BRD4 promote specific cell cycle gene progression by activation of oncogenes (e.g., *MYC, BCL6*, and *TP63)* (60–64) and resistance to apoptosis by upregulating anti-apoptotic family member genes including *BCL2* and the cyclin dependent kinase *CDK6* (63). In addition, BET-family members appear to work co-operatively to control the release of pro-inflammatory cytokines from macrophages (65) and tumor cells (66), and BRD4 might act as a co-activator of transcription mediated by the pro-inflammatory molecule nuclear factor κB (NF-κB) (67, 68). Therefore, BET proteins may contribute with the role of immune cells within the tumor microenvironment in facilitating tumor growth and metastasis, through cytokine release.

### Nuclear Protein in Testes (NUTM1)

Native NUTM1 protein expression is localized to the nucleus and it has been identified in germ cells of the testis (4, 69) and ovary (69), ciliary body (36), germ cell tumors (where protein expression is weak), and *NUTM1*-rearranged tumors (36, 69). While the normal function of the NUTM1 protein is related to spermatogenesis (70), overexpression of *NUTM1* fusion genes leads to nuclear entrapment of the NUTM1 protein where it blocks cell differentiation and induces tumor growth (5, 14, 38, 57). The NES and NLS portions of NUTM1 allows the protein to shuttle between the nucleus and cytoplasm when transgenically expressed in cultured cells (5); hence, the tethering of NUTM1 to chromatin by BDs of BRD4 is critical to BRD4-NUTM1 oncoprotein function (5, 57). The AD1 of NUTM1 binds to and activates EP300, a histone acetyltransferase (44, 71), and this interaction plays a critical role in the oncogenic function of BRD-NUTM1 fusion protein (44, 57, 71, 72).

### Nuclear Receptor Binding SET Domain Protein 3 (NSD3)

The *NSD3* (also known as Wolf-Hirschhorn syndrome candidate 1-like 1, *WHSC1L1*) encodes a histone lysine methyltransferase that belongs to the mammalian NSD protein family of SET domain-containing methyltransferases (i.e., NSD1, NSD2, NSD3). Both NSD3 and NSD2 are known to bind the ET domain of BRD4. The NSD3 protein is considered an enzymatic protein (i.e., histone lysine methyltransferase or HMTases) involved in the methylation of histone lysine marks, regulating chromatin integrity and gene expression (73). Histone marks created by lysine HMTases are associated with either active transcription (e.g., H3K4me or H3K36me2) or repressed transcription (e.g., H3K27me or H2K9me) (74, 75). Only the N-terminus of NSD3, which binds to BRD4, is included in the genetic *NSD3-NUTM1* fusion process, whereas its methyltransferase domain is absent (38, 76, 77). This interaction of BRD4 with NSD3 may be critical to BRD4-NUTM1 oncoprotein function. NSD3 may block differentiation in BRD4-NUTM1 expressing NC cells either through regulation of H3K36 methylation, leading to activation of specific gene expression (38, 43), or through interactions with the histone protein variant macroH2A1 where it represses or activates transcription of specific genes (78, 79). In addition, the *NSD3-NUTM1* fusion oncogene encodes a protein that is necessary and sufficient for the blockade of differentiation in NC (38).

### Zinc Finger Proteins

The ZNF532, ZNF592, ZNF687, and ZMYND8 have been collectively termed “Z4” protein factors. Although understanding of Z4 function is limited, some of them have been shown to play a role in cancer pathogenesis, either as oncogenes or tumor-suppressor genes (45). These zinc finger proteins have been shown to co-localize with BRD4/BRD4-NUTM1 chromatin complex proteins within megadomain regions (43–45). The ZNF532-NUTM1 and ZNF592-NUTM1 resultant fusion proteins form megadomains of hyperacetylated chromatin, similar to those formed by BRD4-NUTM1, suggesting that *ZNF532* and *ZNF592* genes are involved in a common feed-forward regulatory mechanism for megadomain formation that drive propagation of the oncogenic chromatin complex in BRD4-NUTM1 cells (44, 45). Moreover, BRD4-NUTM1-driven foci formation of Z4 factors suggests a mechanism of pathologic sequestration that may alter the normal function of Z4 proteins while enhancing those of BRD4-NUTM1 (45, 71, 80).

### BRD-NUTM1 Protein Function

**Figure 3:**
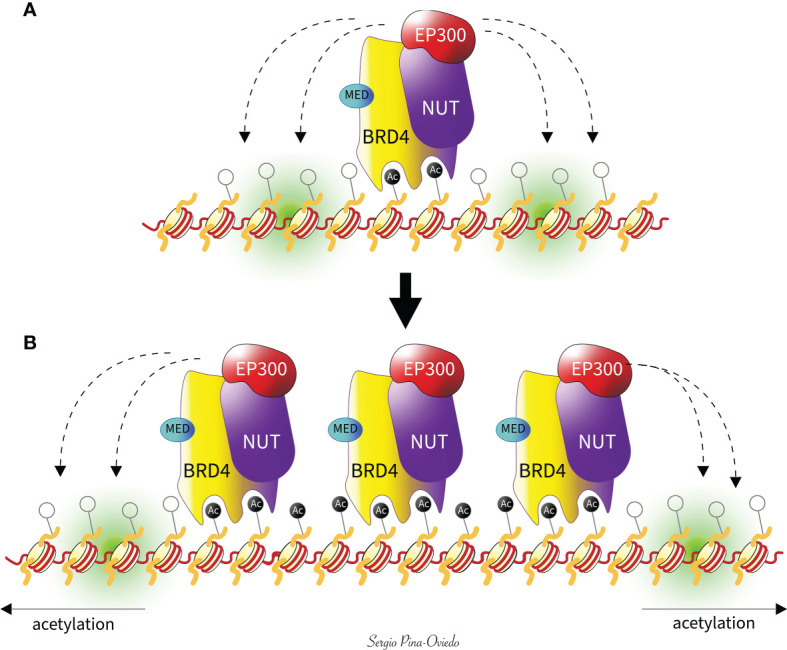
Feed-forward model of megadomain formation. **(A)** BRD4-NUTM1 is tethered to acetylated chromatin by BRD4 bromodomains. EP300 is recruited by the NUTM1 portion of the fusion, leading to increased local histone acetylation and, subsequently, **(B)** a self-perpetuating recruitment of BRD4-NUTM1 complexes. The resultant uncontrolled spreading of BRD4-NUTM1 within the chromatin leads to the formation of megadomains that are typically limited only by topologically associating domain boundaries (not shown). The Mediator subunit 24 (MED) interacts physically with BRD4 as a target and a cofactor of BRD4-NUTM1, to provide another positive reinforcement loop for the establishment of megadomains and their transcriptional activity.

The tethering of NUTM1 to acetylated chromatin by BRD3/BRD4 leads to local chromatin acetylation by recruitment of EP300, resulting in a feed-forward expansion of acetylated chromatin and BRD-NUTM1 chimeric oncoprotein formation over massive genomic domains (megadomains), often filling entire topologically associating domains (5, 64, 71) (**Figure 3**). The number and magnitude of these “megadomains”, measuring from 100 kilobases up to 2 megabases in size, correlate with the characteristic nuclear foci seen in diagnostic patient tumor samples or in cultured NC cells stained with a NUT-specific antibody (5, 42, 64, 71). Additionally, the association of BRD4 to those regions has also been defined by the presence or co-localization of H3K27ac and H3K18ac, and the absence of H3K4m3 (60, 62, 81). In contrast, areas away from the megadomains become hypoacetylated, resulting in transcriptional repression of pro-differentiation genes (64, 72). As a result, these megadomain regions can drive targeted oncogene transcription. *MYC* has been shown to be a downstream oncogene target of BRD4-NUTM1 that blocks NC cellular differentiation and maintains a proliferative state (57). Moreover, there is evidence that BRD4-NUTM1 facilitates acetylation of *TP53* through EP300, leading to its sequestration and inactivation within BRD4-NUTM1 foci (71). Also, it has been found that *TP63*, a *TP53*-related squamous cell-expressed gene, is regulated by BRD4-NUTM1 megadomains in all NC cells tested. Hence, the increased expression of *TP63*, a negative regulator of *TP53*, might represent another mechanism by which BRD4-NUTM1 NC cells evade gate keeper functions of *TP53* (64). Another oncogenic target of BRD4-NUTM1 is the sex-determining region Y-box protein 2 (*SOX2*) which is a transcription factor essential for stem cell self-renewal and pluripotency (82). Although SOX2 expression is normally restricted to stem cells, aberrant overexpression has been linked to its ability to promote tumorigenicity and poorly differentiated morphology (83–86). BRD4-NUTM1 has been shown to drive overexpression of SOX2 in NC cells, which induces an aberrant stem cell-like growth feature (87). Furthermore, *MED24*, a Mediator subunit known to interact physically with BRD4 (43, 63), has been found to participate as both an oncogenic target gene and a cofactor of BRD4-NUTM1 complexes likely to provide another positive reinforcement loop for the establishment of megadomains and their transcriptional activity (64) (**Figure 3**). MED24 plays a role in transcriptional regulation during embryonic development (88), while its post-embryonic role appears to be tissue-specific coactivation of gene expression (89). These findings have been demonstrated in several *in vitro* studies when small interfering RNAs against NUTM1 or small-molecule BET inhibitors, such as JQ1, have been used to knockdown BRD3/4-NUTM1 and NSD3-NUTM1 patient-derived-tumor cells, leading to cellular differentiation and growth arrest (5, 38, 57, 64, 90). This indicates that NUTM1 fusion proteins act to maintain growth and block squamous-cell differentiation, in a mechanism dependent on the targeting of *MYC, SOX2*, *MED24*, and *TP63* genes by BRD3/4-NUTM1 megadomains (10, 44, 57, 87).

Furthermore, wild-type BRD3, NSD3, and other Z4 protein factors (ZNF532, ZNF592, ZNF687 and ZMYND8) interact with BRD4, and its fusion with NUTM1 results in a powerful oncogenic complex. All these rare fusion partners of *NUTM1* are functionally related to BRD4, indicating that the recruitment of NUTM1 to the chromatin through the BET family proteins is necessary in NC pathogenesis (38, 43–45).

## Methods of Diagnosis

A definitive diagnosis of NC requires demonstrating the presence of *NUTM1* gene rearrangement, which can be confirmed by immunohistochemistry with a NUT specific monoclonal antibody (clone C52) (**Figure 1D**). The immunohistochemical stain detects the presence of NUTM1 protein and has been reported as a relatively sensitive (87%) and highly specific (nearly 100%) tool for the diagnosis of NC (69). Diffuse (>50%) and strong nuclear positivity for NUTM1 is considered sufficient evidence for *NUTM1* rearrangement, obviating the need of highly specialized genetic testing (1, 69). An alternative to NUT immunohistochemistry is molecular analysis to detect a *NUTM1* gene rearrangement using fluorescence *in situ* hybridization, reverse-transcriptase polymerase chain reaction, cytogenetics, next generation sequencing, or whole-exome sequencing-based approaches. These methods should be considered if NUT immunohistochemistry is not available or if the result is negative or equivocal, and suspicion of NC is still high (29, 41, 51, 91–93).

## Prognosis and Predictive Factors

The prognosis and outcomes of NC are very dismal with a median survival of 6.5 months (12) and poor response to conventional chemotherapeutic agents or radiotherapy. About 50% of patients present with lymph node involvement or distant metastatic disease (3, 9), frequently seen in lung and bones, and rarely in adrenal glands, brain, bone marrow, and liver (10, 14, 26, 36).

Although not required for diagnosis, molecular techniques can be used to determine the specific *NUTM1* fusion partner which could be of potential prognostic and therapeutic significance. Recently, Chau et al. (12) proposed a prognostic risk classification model for NC survival outcomes based in the largest cohort of NC patients (n = 141) analyzed to date. In this study, they identified three distinct risk groups of patients based on anatomic site and *NUTM1* fusion type, composed by the following: (1) Group A, patients with non-thoracic primary NC and presence of *BRD3*- or *NSD3-NUTM1* fusion, (2) Group B, patients with non-thoracic primary NC and presence of *BRD4-NUTM1* fusion, and (3) Group C, patients with thoracic primary NC regardless of the type of *NUTM1* fusion. Interestingly, *NSD3*- or *BRD3-NUTM1*-positive tumors of non-thoracic origin are associated with significantly better overall survival, followed by the group of non-thoracic primary NC with *BRD4-NUTM1* fusion. On the other hand, those patients with thoracic primary tumors, regardless of the *NUTM1* fusion have worst prognosis than the other subgroups (12).

## Therapeutics

At the current time, there is no standard treatment for this rare and aggressive form of cancer. However, a multimodal approach with aggressive initial surgical resection, systemic chemotherapy, and radiation therapy is currently adopted in clinical practice (11). A variety of chemoradiation therapy regimens have been used including intensive treatments commonly applied in other carcinomas, sarcomas, germ cell tumors, and other solid neoplasms. Some of the chemotherapeutic agents that have been used with some success include cisplatin, taxanes and alkylating agents (9, 11, 24, 33, 94). However, despite rapid response, tumors become treatment-refractory with early progression and poor overall outcome (9, 13).

Targeted therapy using small-molecule BET inhibitors, which are acetyl-lysine histone mimetic drugs, result in depletion of megadomains, proliferation arrest, and cellular differentiation (1, 90). BET inhibitors (e.g., Birabresib aka OTX015/MK-8628, Molibresib aka GSK525762, RO6870810, ODM-207, and NEO2734) have shown activity but no obvious survival benefit (3, 95–100), most likely due to toxicity effects (i.e., severe thrombocytopenia, gastrointestinal symptoms, anemia, and fatigue), limiting its use (3, 96, 98). However, by the time of the writing of this manuscript, there is an ongoing clinical trial where pediatric patients with solid tumors, brain tumors and lymphoma are being enrolled on a Phase I research study to evaluate the use of BET inhibitors (known as BMS-986158 and BMS-986378) in those patients described above and as possible treatments for NUT carcinoma in children. Both of these drugs are currently still being studied in adult patients (101). Other preclinical studies have shown that the *BRD4-NUTM1* fusion gene is associated with global decreased histone acetylation and transcriptional repression of genes required for differentiation. Some *in vitro* and xenograft models have shown that this acetylation can be restored with histone deacetylase (HDAC) inhibitors such as Vorinostat, resulting in global increase in histone acetylation, squamous differentiation, and growth arrest (72, 102). However, the use of HDAC inhibitors has been limited due to the toxicity effects like those seen with BET inhibitors (72). Both of these novel targeted agents hold great promise, either alone or in combination with chemotherapy (37). In particular, preclinical studies have highlighted that BET inhibitors show synergism with immune checkpoint modulators (103–105).

Currently, patients can be enrolled into the International NUT Midline Carcinoma Registry (http://www.nmcregistry.org) which follows patient’s outcomes and may direct them to the institution running these trials (1, 35, 37). This international registry was originally established in 2010 and was created to raise awareness and disseminate the most updated information about NC, provide pathologic review to assist in the diagnosis of NC, and collect clinical data and response to treatment. This has allowed the creation of a repository of clinical specimens that will support future research (106).

## Conclusions

In summary, this an overview of NUT carcinoma with a brief discussion of the main epidemiologic and clinicopathologic features, along with the main molecular genetics/epigenetics findings and therapeutics for this tumor. Although several *NUTM1* translocations have been found to be associated with NUT carcinoma, to the best of our knowledge, up to this day there is no known specific etiology for the *NUTM1* translocation. In addition, given the rarity and relatively recent description of this entity, studies about the complete mutational landscape of NUT carcinoma are not yet available. Therefore, additional mutations that could play a role in oncogenesis are not well studied or known at this point in time. The awareness of this deadly tumor and the understanding of the underlying molecular mechanisms, genetics, and epigenetics will be helpful in future research for the development of novel targeted therapies.

## Author Contributions

Conceptualization: VM and SP-O. All authors VM, KS, and SP-O drafted, revised, and approved the submitted version of this manuscript.

## Conflict of Interest

The authors declare that the research was conducted in the absence of any commercial or financial relationships that could be construed as a potential conflict of interest.

## Publisher’s Note

All claims expressed in this article are solely those of the authors and do not necessarily represent those of their affiliated organizations, or those of the publisher, the editors and the reviewers. Any product that may be evaluated in this article, or claim that may be made by its manufacturer, is not guaranteed or endorsed by the publisher.
